# XVIII Reunión Post-ECTRIMS: Revisión de las Novedades Presentadas en el Congreso ECTRIMS 2025 (Parte II)

**DOI:** 10.31083/RN55658

**Published:** 2026-08-20

**Authors:** Óscar Fernández, Xavier Montalban, Adrián Arés, Eduardo Agüera, Yolanda Aladro, Ana Alonso, Rafael Arroyo, Luis Brieva, Carmen Calles, Ana Belén Caminero, Tamara Castillo-Triviño, Lucienne Costa-Frossard, Sara Eichau, Lamberto Landete, Miguel Llaneza, Sara Llufriu, José E. Meca-Lallana, Virginia Meca-Lallana, Enric Monreal, Ester Moral, Celia Oreja-Guevara, José María Prieto, Lucía Romero-Pinel, Àlex Rovira, Alfredo Rodríguez-Antigüedad

**Affiliations:** ^1^Departamento de Farmacología, Facultad de Medicina, Universidad de Málaga, 29016 Málaga, España; ^2^Instituto de Investigación Biomédica de Málaga (IBIMA), Hospital Universitario Regional de Málaga, Universidad de Málaga, 29016 Málaga, España; ^3^Centro de Esclerosis Múltiple de Cataluña y Departamento de Neurología, Hospital Universitario Vall d’Hebron, 08035 Barcelona, España; ^4^Universitat Autònoma de Barcelona, 08193 Bellaterra, España; ^5^Servicio de Neurología, Complejo Asistencial Universitario de León, 24008 León, España; ^6^Servicio de Neurología, Hospital Reina Sofía, 14004 Córdoba, España; ^7^Servicio de Neurología, Hospital Universitario de Getafe, 28905 Getafe, España; ^8^Unidad de Esclerosis Múltiple, Servicio de Neurología, Hospital Regional Universitario de Málaga, 29010 Málaga, España; ^9^Servicio de Neurología, Hospital Universitario Quirónsalud, 28223 Pozuelo de Alarcón, España; ^10^Universitat de Lleida, 25198 Lleida, España; ^11^Hospital Universitari Arnau de Vilanova, Institut de Recerca Biomèdica de Lleida (IRBLleida), 25198 Lleida, España; ^12^Servicio de Neurología, Hospital Universitario Son Espases, 07120 Palma de Mallorca, España; ^13^Servicio de Neurología, Complejo Asistencial de Ávila, 05071 Ávila, España; ^14^Servicio de Neurología, Hospital Universitario Donostia, Grupo de Neuroinmunología, IIS Biogipuzkoa, 20014 Donostia, España; ^15^CSUR de Esclerosis Múltiple, Hospital Ramón y Cajal, 28034 Madrid, España; ^16^Servicio de Neurología, Hospital Universitario Virgen Macarena, 41009 Sevilla, España; ^17^Servicio de Neurología, Hospital Universitario Doctor Peset, 46017 Valencia, España; ^18^Servicio de Neurología, Hospital Universitario Central de Asturias, 33011 Oviedo, España; ^19^Unidad de Neuroinmunología y Esclerosis Múltiple, Hospital Clínic de Barcelona e IDIBAPS, 08036 Barcelona, España; ^20^CSUR Esclerosis Múltiple, Unidad de Neuroinmunología Clínica, Servicio de Neurología, Hospital Clínico Universitario Virgen de la Arrixaca (IMIB-Arrixaca), 30120 El Palmar, España; ^21^Cátedra de Neuroinmunología Clínica y Esclerosis Múltiple, Universidad Católica San Antonio (UCAM), 30107 Guadalupe, España; ^22^Servicio de Neurología, Hospital Universitario de la Princesa, 28006 Madrid, España; ^23^Servicio de Neurología, Complejo Hospitalario Universitario Moisès Broggi, 08970 Barcelona, España; ^24^Servicio de Neurología, Hospital Clínico San Carlos, Instituto de Investigación Sanitaria San Carlos (IdISSC), 28040 Madrid, España; ^25^Departamento de Medicina, Facultad de Medicina, Universidad Complutense de Madrid (UCM), 28040 Madrid, España; ^26^Servicio de Neurología, Instituto de Investigación Sanitaria de Santiago de Compostela (IDIS), 15706 Santiago de Compostela, España; ^27^Hospital Universitari de Bellvitge-Institut d'Investigació Biomèdica de Bellvitge (IDIBELL), 08908 L’Hospitalet de Llobregat, España; ^28^Sección de Neuroradiología, Departamento de Radiología, Hospital Universitari Vall d'Hebron, 08035 Barcelona, España; ^29^Universitat Autònoma de Barcelona, 08193 Barcelona, España; ^30^Servicio de Neurología, Hospital Universitario Cruces, 48903 Barakaldo, España

**Keywords:** ECTRIMS, esclerosis múltiple, post-ECTRIMS, ECTRIMS, multiple sclerosis, post-ECTRIMS

## Abstract

La XVIII edición de la reunión post-ECTRIMS (*European Committee for Treatment and Research in Multiple Sclerosis*) se celebró los días 17 y 18 de octubre de 2025 en Madrid, congregando a neurólogos españoles especializados en esclerosis múltiple (EM), quienes presentaron los avances más relevantes discutidos en el congreso ECTRIMS, celebrado días antes en Barcelona. El objetivo de esta edición fue presentar las novedades en EM sobre actualización de criterios diagnósticos, técnicas avanzadas de neuroimagen, el papel del virus de Epstein-Barr, mecanismos de remielinización y biomarcadores. En el ámbito terapéutico se abordaron tratamientos emergentes como nuevos anticuerpos monoclonales y células T con receptor de antígeno quimérico (CAR-T, *chimeric antigen receptor T cell*), el manejo en pacientes de edad avanzada y los riesgos asociados a las terapias de alta eficacia. Este artículo corresponde al segundo de una serie de dos artículos (I y II). En él se revisan datos recientes sobre la progresión de la enfermedad y sobre el concepto progresión independiente de brotes (PIRA, *progression independent of relapse activity*), incluyendo la definición, de entre todas las publicadas, que conduce a una incidencia y persistencia equilibradas en las tasas de PIRA. Se repasan ciertos aspectos neuropatológicos y de imagen, y respecto a los biomarcadores séricos, se insiste en que los neurofilamento de cadena ligera (NfL) son un marcador de daño agudo no específico de EM, y que el reto está en plantear un valor diferenciador de otras enfermedades. También se discute sobre si la baja sensibilidad de los NfL para predecir progresión radique en el z-score que se maneja en la práctica clínica, y sobre la utilidad de la proteína acídica fibrilar glial (GFAP) en combinación con los NfL para refinar el daño progresivo crónico. La importancia de la edad en EM es indiscutible. El paciente con EM de edad avanzada es un paciente con un mayor número de comorbilidades y más vulnerable a ciertos tratamientos. La menopausia es una transición fisiológica normal pero esperada del envejecimiento de la mujer que puede desencadenar o agravar ciertos problemas de salud, y cuyos síntomas no deberían confundirse con un empeoramiento de la enfermedad. En cuanto al deterioro cognitivo en EM, se recogen las novedades y opiniones de los expertos que podrían ayudar en una mejor identificación y monitorización. Gran parte de este segundo artículo se centra en resumir lo más destacado en cuanto a terapias emergentes y expectativas de tratamiento en enfermedades relacionadas, sin olvidar los riesgos asociados a las terapias de muy alta eficacia y las posibles estrategias de prevención del riesgo. Por último, como datos importantes que se trasladan a la práctica clínica, destaca la notable mejora en el diagnóstico con los nuevos criterios de McDonald 2024, a pesar de las barreras de acceso a las pruebas paraclínicas en determinadas poblaciones. En conjunto, los datos revisados, junto con los recogidos en la parte I, reflejan el esfuerzo investigador por avanzar en el conocimiento de la enfermedad desde múltiples perspectivas complementarias, y que están transformando la atención del paciente con EM.

## Abstract (en)

The 18th edition of the post-European Committee for Treatment and Research in Multiple Sclerosis (ECTRIMS) meeting was held on 17–18 October 2025 in Madrid, bringing together Spanish neurologists specialising in multiple sclerosis (MS), who presented the most relevant advances discussed at the ECTRIMS congress held a few days earlier in Barcelona. The aim of this edition was to present the latest developments in MS regarding the update of diagnostic criteria, advanced neuroimaging techniques, the role of Epstein-Barr virus, remyelination mechanisms and biomarkers. In the therapeutic field, emerging treatments were addressed, such as new monoclonal antibodies and chimeric antigen receptor T cells (CAR-T), the management of elderly patients and the risks associated with high-efficacy therapies. This article is the second of a two-part series (I and II). The article reviews recent data on disease progression and on the concept of progression independent of relapse activity (PIRA), including the definition, among those published, that leads to balanced incidence and persistence in PIRA rates. Certain neuropathological and imaging aspects are reviewed, and, with regard to serum biomarkers, it is emphasised that neurofilament light chain (NfL) is a marker of acute damage that is not specific to MS, and that the challenge lies in establishing a value that differentiates it from other diseases. It is also discussed whether the low sensitivity of NfL for predicting progression lies in the z-score used in clinical practice, and the usefulness of glial fibrillary acidic protein (GFAP) in combination with NfL to refine the assessment of chronic progressive damage. The importance of age in MS is indisputable. The elderly patient with MS has a greater number of comorbidities and is more vulnerable to certain treatments. Menopause is a normal but expected physiological transition of female ageing that may trigger or aggravate certain health problems, and whose symptoms should not be confused with worsening of the disease. As for cognitive impairment in MS, the latest developments and expert opinions that could help improve its identification and monitoring are summarised. Much of this part II article focuses on summarising the key advances in emerging therapies and treatment prospects in related diseases, without overlooking the risks associated with very high-efficacy therapies and the possible risk-prevention strategies. Finally, as important findings that translate into clinical practice, the notable improvement in diagnosis with the new 2024 McDonald criteria stands out, despite the barriers to accessing paraclinical tests in certain populations. Overall, the data reviewed, together with those gathered in part I, reflect the research effort to advance the understanding of the disease from multiple complementary perspectives, which is transforming the care of patients with MS.

## 1. Introducción

Los días 17 y 18 de octubre de 2025 tuvo lugar la XVIII edición de la reunión Post-ECTRIMS (*European Committee for Treatment and Research in Multiple Sclerosis*). Como cada año, varios neurólogos expertos en esclerosis múltiple (EM) en España presentaron un resumen de las principales novedades del congreso ECTRIMS, celebrado en Barcelona los días 24–26 de septiembre. Los resúmenes de dichas ponencias se presentan en dos artículos (I y II).

La selección de las ponencias presentadas se realizó siguiendo un enfoque temático coherente con el utilizado en ediciones previas del Post-ECTRIMS, con el objetivo de garantizar una cobertura equilibrada y dar prioridad a las líneas de investigación con un posible mayor impacto en la práctica clínica.

Este segundo artículo (II) aborda las novedades en progresión independiente de la actividad de brotes (PIRA), la importancia de la edad en EM y cómo la mayor esperanza de vida de los pacientes, atribuible a los avances terapéuticos, ha hecho más visibles las comorbilidades y la importancia de tenerlas en cuenta a la hora de planificar el tratamiento. Se discute sobre la menopausia, que pese a ser una fase de gran influencia en la mujer, es una etapa fisiológica poco estudiada en mujeres con EM y cuyos síntomas pueden confundirse con un empeoramiento de la enfermedad y complicar la atención de las pacientes. También se presentan las novedades que guiarán al neurólogo en una mejor identificación y monitorización del deterioro cognitivo. Gran parte de este segundo artículo se centra en revisar lo más destacado en cuanto a las terapias emergentes, y las expectativas de tratamiento en enfermedades relacionadas, sin olvidar los riesgos asociados a las terapias de muy alta eficacia y las posibles estrategias de prevención del riesgo. Por último, se destacan ciertos aspectos clínicos importantes para llevarse a la práctica clínica en cuanto a diagnóstico, pronóstico y personalización del tratamiento.

## 2. Progresión en EM y Definición de PIRA: Aspectos Neuropatológicos, Biomarcadores e Imagen

La progresión es un rasgo distintivo de la EM, que comienza en fases muy iniciales, y probablemente de una forma más intensa que cuando se detecta clínicamente [1]. La progresión impacta en neuronas de tractos largos, quizás más demandantes o susceptibles [1].

Dentro de las características radiológicas de la progresión, las lesiones de borde ancho (BRLs) detectadas en la tomografía por emisión de positrones (PET), marcadas por un extenso anillo de células mieloides, se presentan como un potencial marcador de imagen de progresión rápida [2]. La patogenia de la sustancia gris (SG) podría ser un actor clave en la patogénesis de la progresión, destacándose los recientes hallazgos de una relación entre la captación cortical de la proteína translocadora (TSPO, *translocator protein*) en la PET y la progresión clínica [3].

En cuanto a los biomarcadores séricos, se insiste en que los neurofilamento de cadena ligera (NfL) son un marcador de daño agudo no específico de EM; por tanto, el reto está en plantear un valor diferenciador de otras enfermedades con un patrón de muerte neuronal muy diferente. También es posible que la “baja” sensibilidad de los NfL para predecir progresión radique en el z-score que se maneja en la práctica clínica: un z-score >2 indica lesión focal [4] pero un z-score <1,5 o <1,0 predice la progresión confirmada de la discapacidad (CPD) [5] y la PIRA a 6 años [6]. A ello se añade que los valores z-score de NfL, no los valores absolutos, predicen futuros brotes [7] pero solo se asocian con progresión de la discapacidad a nivel grupal, no individual [8].

Es probable que el cierto desconocimiento de la biología del astrocito impida entender bien el papel de la proteína acídica fibrilar glial (GFAP), pero podría ser de utilidad en combinación con los NfL para refinar el daño progresivo crónico en los casos con valores de z-score NfL <1,5 [4].

### Definición de PIRA

**Fig. 1.**
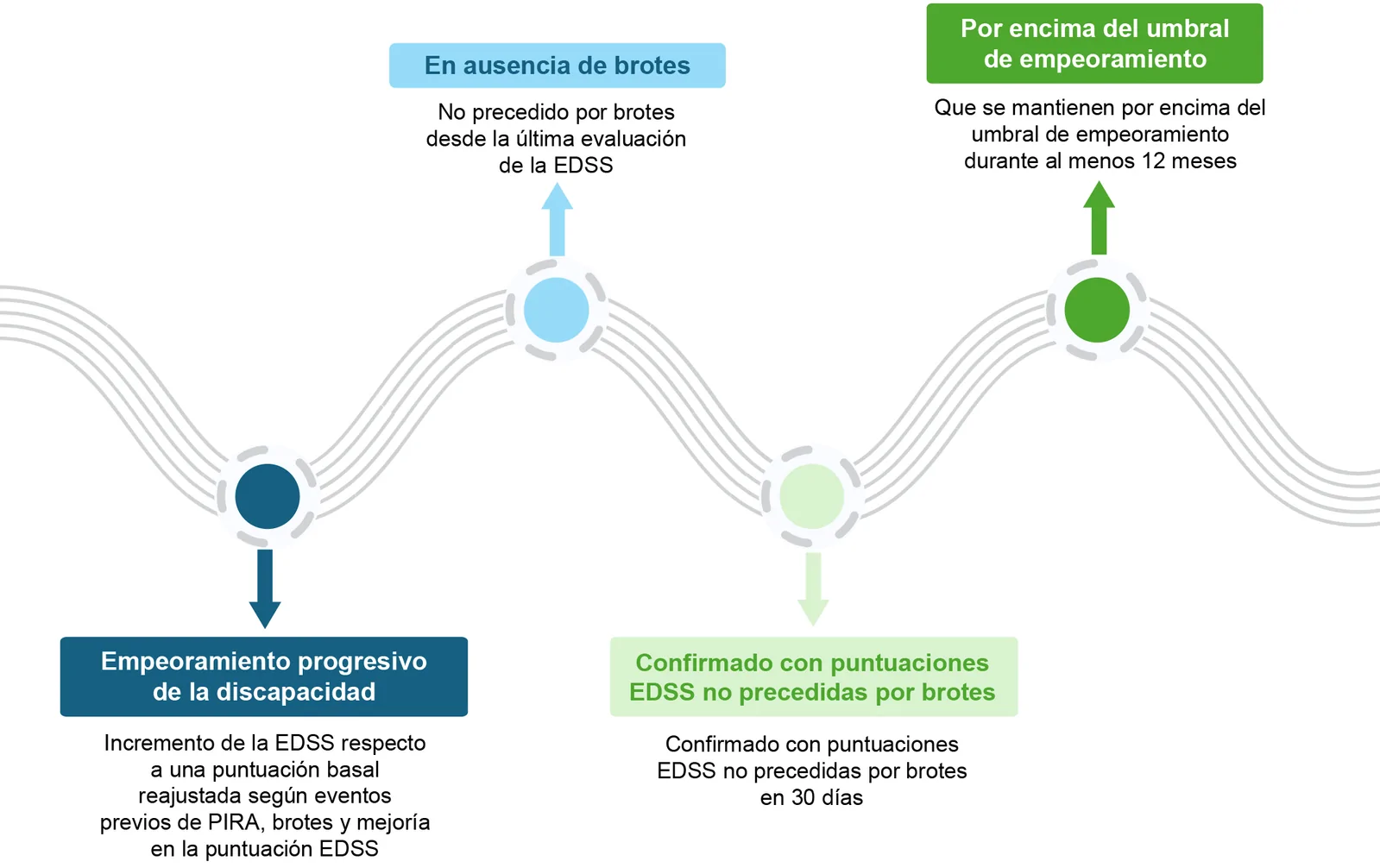
**Definición de PIRA que lleva a una incidencia y persistencia equilibradas en las tasas de PIRA**. EDSS, escala ampliada del estado de discapacidad; PIRA, progresión independiente de la actividad de brotes.

PIRA se reconoce como factor clave de discapacidad en EM, aunque es un concepto elusivo por la incertidumbre sobre su definición, mecanismos subyacentes y cuantificación. La falta de consenso en su definición genera una variabilidad en las tasas de PIRA que oscilan entre un 6–28% [5] y en el efecto del tratamiento en ensayos clínicos según la definición empleada [6]. Un análisis reciente del registro MSBase con datos del 2004–2023, identificó hasta 360 definiciones de PIRA [7]. La Fig. 1 muestra la definición de entre todas que llevó a una incidencia y persistencia equilibradas en las tasas de PIRA [7].

Uno de los principales desafíos en ensayos clínicos con terapias de alta eficacia son las estimaciones sesgadas de PIRA por el efecto del tratamiento en la reducción de los brotes, recomendándose el ajuste con el empeoramiento asociado a brotes (RAW) para eliminar este enmascaramiento [8]. En la práctica clínica, además de la mencionada falta de consenso en la definición, destacan como principales problemas la falta de estandarización en la frecuencia de visitas y evaluaciones, y la omisión habitual de la actividad en resonancia magnética (RM). Además, la afectación en otros dominios motores, detectada mediante la prueba cronometrada de 25 pies (T25-FW*, Timed 25-Foot Walk*) y la prueba de los 9 agujeros (9-HPT, *Nine-Hole Peg Test*), puede ser relevante y particularmente útil en detección de PIRA a 12 y 24 semanas [9]. El PIRA cognitivo, definido como deterioro cognitivo en ausencia de brotes entre evaluaciones ni en los 9 meses posteriores representa la mayoría del deterioro cognitivo en pacientes con EM remitente-recurrente (EMRR) [10], ampliando la comprensión del deterioro cognitivo en la enfermedad.

En cuanto a las medidas de RM, la tasa de atrofia cerebral acelerada es superior en pacientes con PIRA frente a pacientes estables [11]. En pacientes con formas primarias progresivas (PP) no inflamatorias según parámetros clínicos y radiológicos, solo los niveles séricos de la proteína similar a la quitinasa 3 tipo 1 (CHI3L1, chitinase-3-like protein 1) permanecieron asociados con futuros cambios en la escala ampliada del estado de discapacidad (EDSS, *Expanded Disability Status Scale*) frente a NfL y GFAP [12].

En definitiva, para entender el fenómeno PIRA hemos de ampliar la visión e integrar la afectación en todos los dominios clínicos junto con las medidas en RM y los biomarcadores.

## 3. Edad en EM y Aparición Tardía de la Enfermedad

Entre los mecanismos biológicos del envejecimiento en la EM, destaca el descubrimiento del gen *XRCC5* por su vínculo con el envejecimiento celular y la susceptibilidad a la enfermedad [13]. Otra línea de investigación es el estudio de un subgrupo de células B asociadas a la edad (ABC), que proliferan con la edad y se localizan en los folículos meníngeos [13]. Es sabido que con la edad el sistema inmune cambia, los linfocitos B envejecen y se reduce la capacidad de remielinización de los oligodendrocitos y astrocitos, de ahí la importancia de considerar la edad y los mecanismos biológicos asociados en el desarrollo clínico de futuros fármacos [13].

**Fig. 2.**
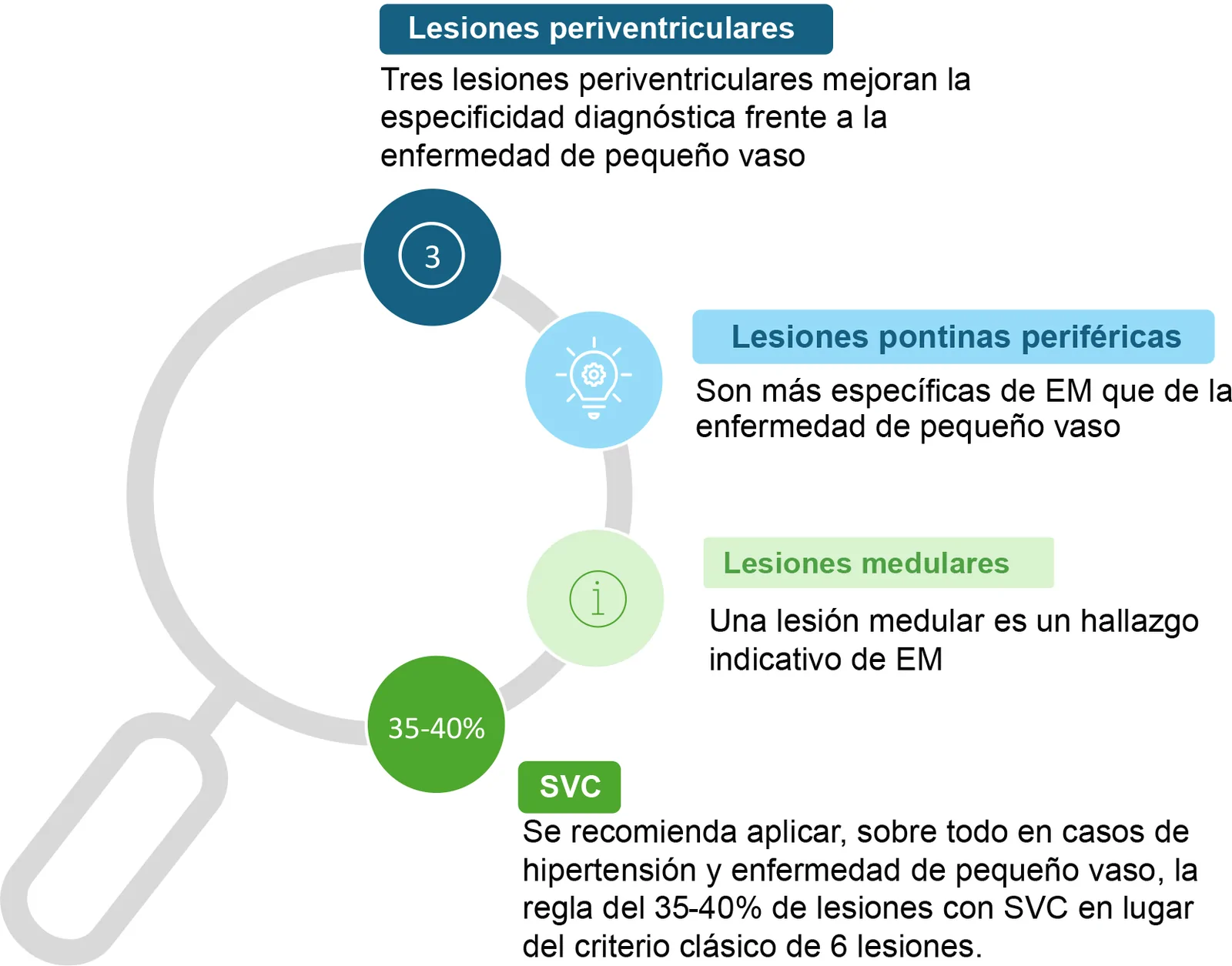
**Recomendaciones en el paciente ≥50 años (Nuevos criterios de McDonald 2024, [14])**. EM, esclerosis múltiple; SVC, signo de la vena central.

Los pacientes mayores de 50 años constituyen una población especial en el algoritmo de aplicación de los nuevos criterios de McDonald 2024 [14], con ajustes relevantes (Fig. 2). A esto se une el aumento de los casos de EM de inicio tardío que complica el manejo terapéutico debido al aumento de los riesgos asociados al tratamiento. En estos pacientes, la administración de tratamientos de alta eficacia suele ser más tardía y menos frecuente [15], y aunque la edad no constituye por sí misma una contraindicación para estos fármacos, la evaluación del balance riesgo/beneficio debe individualizarse considerando factores como las comorbilidades, procesos infecciosos y neoplasias. Esta decisión se ve además dificultada por la falta de evidencia sólida derivada de la infrarrepresentación de esta población en ensayos clínicos [16]. Como la estrategia terapéutica individualizada también es necesaria en personas mayores de 50 años, una posible opción es el inicio de terapias depletoras de células B, como los anti-CD20, en casos seleccionados [15].

## 4. Manejo del Paciente de Edad Avanzada

El aumento de la supervivencia, de la edad media de los pacientes con EM, de los diagnósticos de inicio tardío o muy tardío y los cambios en los mecanismos patogénicos, sitúan al paciente de edad avanzada dentro de los retos del tratamiento. A esto se une un descenso de la eficacia del tratamiento inmunomodulador con la edad [17], la falta de evidencia de los efectos del tratamiento en esta población fruto de la no inclusión en los ensayos clínicos [18], el aumento del riesgo de infecciones [19] y de neoplasias [20], y el sesgo en la elección del tratamiento debido a un menor acceso al tratamiento de alta eficacia, o a recibir tratamiento en los casos con formas primarias progresivas [21]. Tampoco hay evidencia suficiente que avale la discontinuación del tratamiento en esta población de pacientes, y por tanto habría que plantear estrategias en la vía de la desescalada terapéutica [22].

El manejo de la enfermedad en el paciente de edad avanzada debería ser holístico [22,23]. Es fundamental considerar los aspectos del estilo de vida del paciente, especialmente el ejercicio, y fomentar estrategias preventivas como la vacunación o la participación en programas de cribado de cáncer, sin olvidar el entorno social del paciente que puede limitar el acceso a los servicios de salud, tratamiento o vida saludable [22,23]. Estos pacientes suelen enfrentar múltiples barreras para acceder a una atención de calidad—desde un diagnóstico más complejo, vías digitales o de acceso poco amigables, y un mayor riesgo asociado al tratamiento. Sin embargo, todo paciente con EM tiene derecho a acceder al tratamiento más efectivo posible lo antes posible, independientemente de su edad [22].

Se comentó que medir la edad biológica podría ayudar en la toma de decisiones terapéuticas en esta población [24], teniendo en cuenta que los pacientes con EM presentan un envejecimiento acelerado y un acortamiento precoz de los telómeros, observable incluso en edad pediátrica [25]. La senoterapéutica, dirigida a eliminar las células senescentes o frenar su actividad muestra resultados interesantes sobre cómo el ejercicio [26] y la dieta mediterránea [27] pueden ser beneficiosos para el mantenimiento de la longitud de los telómeros. Estas estrategias deberían implementarse precozmente para crear reserva, sin esperar a la edad avanzada.

### Comorbilidad en EM

La mayor esperanza de vida de los pacientes, atribuible a los tratamientos actuales de alta eficacia y muy alta eficacia, ha hecho más visibles las comorbilidades asociadas al envejecimiento. Por ello, es importante tenerlas en cuenta a la hora de planificar el tratamiento, sobre todo por razones de seguridad, ya que ciertos fármacos pueden agravar alteraciones hepáticas, tiroideas y cardiovasculares [28], siendo necesario un seguimiento más estrecho y personalizado.

En cuanto a la menopausia, representa una fase de gran influencia en la mujer que, paradójicamente, es una de las etapas fisiológicas menos estudiadas en mujeres con EM. Durante este periodo aparecen síntomas como fatiga, trastornos urinarios o insomnio, que pueden confundirse con un empeoramiento de la enfermedad y complicar la atención de las pacientes [29]. En mujeres sintomáticas cuidadosamente seleccionadas, tras valoración individual (edad, tiempo desde menopausia, comorbilidades/contraindicaciones, etc.) la terapia hormonal sustitutiva (THS) puede ofrecer un balance beneficio-riesgo favorable y mejorar la calidad de vida.

## 5. Hacia una Mejor Identificación y Monitorización del Deterioro Cognitivo en EM 

**Fig. 3.**
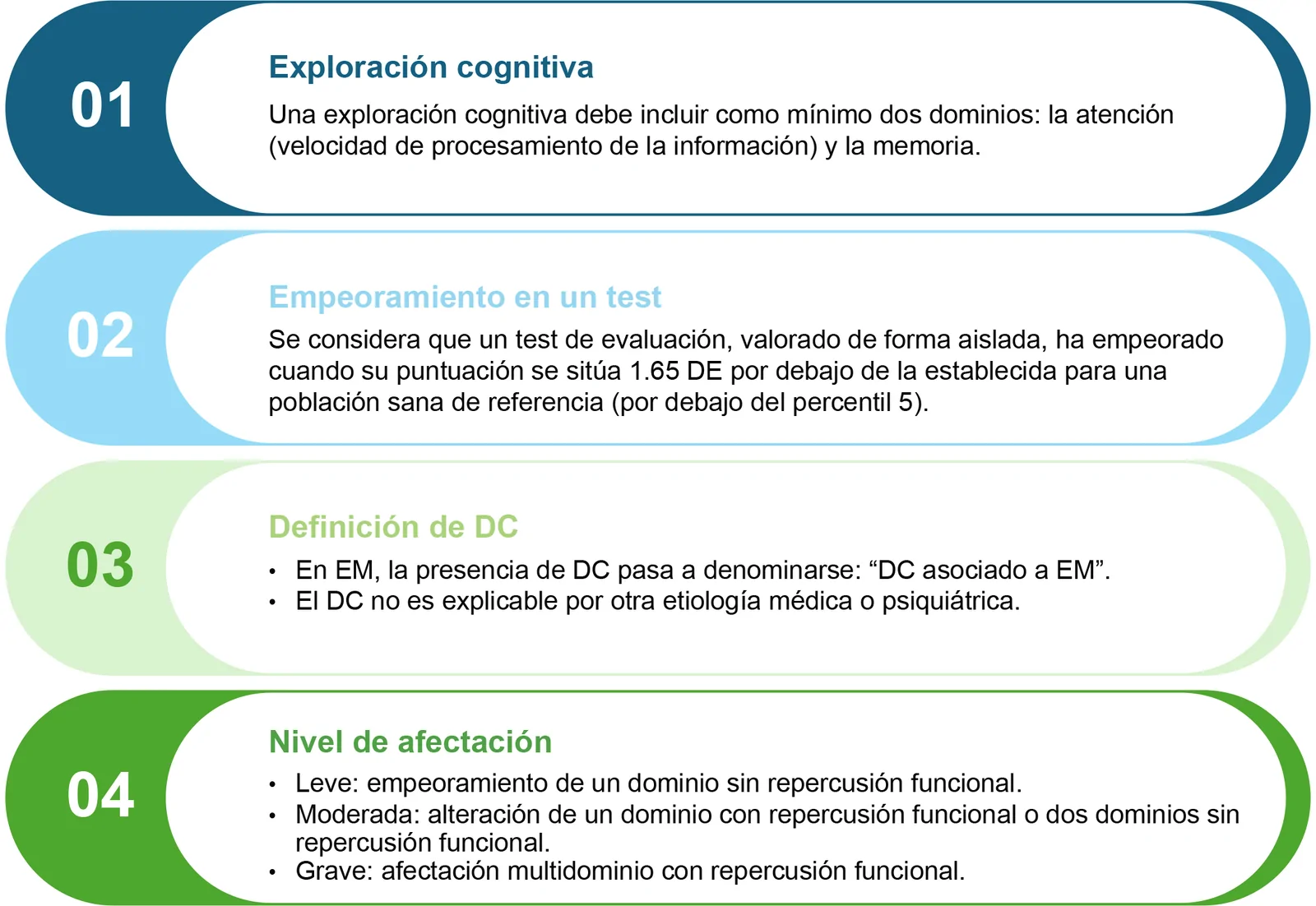
**Nuevas guías diagnósticas de deterioro cognitivo en EM: resultados preliminares presentados en ECTRIMS 2025**. DE, desviación estándar; DC, deterioro cognitivo; ECTRIMS, European Committee for Treatment and Research in Multiple Sclerosis.

La prevalencia del deterioro cognitivo (DC) en EM varía entre el 65–75%, en función de los estudios, al igual que su patrón y grado de afectación; en cualquier caso, las consecuencias laborales, familiares y socio-sanitarias son deletéreas para el paciente. Hasta ahora, el diagnóstico ha sido dicotómico centrándose en la ausencia/presencia de DC sin considerar su gravedad, clasificando conjuntamente a pacientes con afectación leve y grave, pese a sus diferentes necesidades. Para abordar esta limitación, el grupo IMSCOGS (*International MS Cognition Society*) ha desarrollado unas nuevas guías diagnósticas mediante metodología Delphi que abordan la definición de DC, su progresión y las herramientas para evaluarlo (Fig. 3).

Los ensayos clínicos INFORMS [30], ASCEND [31] y DECIDE [32] han mostrado que de forma paradójica y debido al efecto aprendizaje, la puntuación obtenida en los test Paced Auditory Serial Addition Test (PASAT-3) y Symbol Digit Modalities Test (SDMT) mejoran con el tiempo mientras que variables como la puntuación EDSS y atrofia en RM empeoran. Por otra parte, la correlación basal existente entre la puntuación en los tests cognitivos y las variables clínicas y radiológicas desaparece con el tiempo [33]. Este hallazgo es determinante en práctica clínica y confirma que son necesarios nuevos tests cognitivos con mayor sensibilidad al cambio longitudinal. En este contexto, cabe preguntarse cómo definir un cambio cognitivo longitudinal relevante y qué variable utilizar. El análisis del lenguaje se postula como la variable más útil en este sentido, dado que su alteración es muy prevalente, su evaluación escapa al efecto aprendizaje [34], está estrechamente relacionado con la velocidad de procesamiento de la información [35], que constituye el principal déficit en EM, y su fundamento fisiopatológico es robusto, ya que la red del lenguaje es extraordinariamente extensa y se vería afectada desde el inicio por el síndrome de desconexión, típico de EM por la localización subcortical de las lesiones [36].

## 6. Terapias Emergentes en EM: Nuevas Evidencias de Estudios en Fases Tempranas

### 6.1 BTKi

Entre los inhibidores de la tirosina quinasa de Bruton (BTKi, *Bruton’s Tyrosine Kinase inhibitors*) en investigación, algunos podrían integrarse en el arsenal terapéutico de la EM aunque aún es pronto para definir su posicionamiento. Los posibles escenarios de uso serían [37]: (1) tolebrutinib en formas no focales (progresivas y PIRA); (2) fenebrutinib y remibrutinib en formas remitentes; (3) cualquier BTKi como tratamiento secuencial tras alta eficacia/CD20 o combinado con alta eficacia/CD20 en ciertos casos de PIRA.

Destacan los resultados de fenebrutinib en la extensión abierta del estudio fase 2 FENopta [38]. En dicho estudio, el fármaco fue detectable en el líquido cefalorraquídeo (LCR) de 11 pacientes, y se observó una supresión marcada de la actividad radiológica, un 80% de pacientes en estado no evidencia de actividad de la enfermedad (NEDA, *no evidence of disease activity*)-3, un 92% de los pacientes sin brotes, y un 98% libres de progresión de la discapacidad confirmada a las 24 semanas. Asimismo, se observó una reducción en los niveles de NfL a las 48 semanas, y una disminución sostenida de linfocitos B e inmunoglobulinas sin un aumento del riesgo de infecciones tras dos años de seguimiento. No obstante, estos resultados proceden de un diseño abierto, sin grupo comparador, por lo que deben interpretarse con cautela, y, además, plantea dudas sobre el riesgo de la administración secuencial tras agentes anti-CD20.

### 6.2 Anticuerpos Monoclonales

CD19 representa una diana terapéutica de interés en EM, con varios argumentos a su favor [39]: los plasmablastos y las células plasmáticas expresan CD19 y CD138, a diferencia del resto de las células de la estirpe B. Los plasmablastos, además, son un tipo celular predominante en las fases activas de la EM, que intervienen en la síntesis intratecal de IgG, rotura de la barrera hematoencefálica (BHE) y en la actividad de la enfermedad. Los plasmablastos pueden producir anticuerpos que dañan la mielina y además las células plasmáticas que expresan CD138 se han asociado con la progresión de la enfermedad [40].

En modelos animales, los anticuerpos anti-CD19 han demostrado producir una depleción de células B más profunda y duradera que los anti-CD20 [41], y en pacientes con EMRR, el anticuerpo anti-CD19 inebilizumab redujo las lesiones nuevas/aumentadas en T2 y las realzadas con gadolinio (Gd) [42]. Se propone realizar estudios de no inferioridad de inebilizumab frente a los anti-CD20 clásicos, analizando en LCR la depleción de células B, y el comportamiento de plasmablastos, células plasmáticas y componentes humorales.

Foralumab es un anticuerpo anti-CD3 de administración nasal. Los resultados preliminares de un estudio con 10 pacientes sugieren que el fármaco podría atenuar la inflamación microglial y estabilizar la progresión clínica en EM secundaria progresiva no activa [43], aunque se requieren estudios más robustos para confirmar estos hallazgos.

Frexalimab parece bloquear la vía CD40/CD40L sin depleción linfocitaria. Los resultados de la extensión del estudio fase 2 en EM recurrente mostraron una reducción del 89% en la aparición de nuevas lesiones T1-Gd+ y una disminución sostenida de los brotes a lo largo de 2,5 años de seguimiento [44]. Además de reducir los niveles plasmáticos de NfL y quimocina CXCL13 (*C-X-C motif chemokine ligand 13*), el fármaco también actúa sobre el marcador de activación de células T sCD27, y sobre el receptor soluble activador expresado en células mieloides 2 (sTREM2, *soluble triggering receptor expressed on myeloid cells 2*) [45].

### 6.3 Terapia Celular CAR-T

La evidencia disponible, aunque limitada, muestra las ventajas del mecanismo de acción de la terapia de células T con receptor de antígeno quimérico (CAR-T, *chimeric antigen receptor T cell*) y activadores biespecíficos de células T (TCE, *T-cell engagers*), que utilizan linfocitos T como células efectoras para interrumpir la colaboración B-T en la enfermedad autoinmune [46].

Resultados preliminares en fase 1 de terapia CAR-T anti-CD19 en 4 pacientes con EM progresiva han mostrado un perfil de seguridad aceptable sin episodios de síndrome de liberación de citoquinas, síndrome neurológico asociado a células inmunoefectoras (ICANS) ni otros acontecimientos adversos graves. Además, se ha detectado una depleción completa de linfocitos B, y una reconstitución de hasta un 95% de linfocitos B naive (CD19^+^CD27^–^IgD^+^) con un 0–1% de linfocitos B de memoria [47].

## 7. Nuevos Hallazgos con los Anti-CD20 Clásicos

La Tabla 1 (Ref. [48,49,50,51]) presenta los resultados de nuevos ensayos clínicos con los clásicos anti-CD20. En conjunto, los resultados muestran que ocrelizumab 600 mg tuvo un efecto significativo en la progresión de la discapacidad de pacientes con esclerosis múltiple primaria progresiva (EMPP), mayores y con una enfermedad más avanzada [48]; sin embargo, cuando se administró en dosis elevadas no logró reducir la progresión de la discapacidad [49]. En población pediátrica, ocrelizumab se mostró no inferior a fingolimod en controlar los brotes y es superior a fingolimod en reducir la actividad radiológica [50].

**Tabla 1.** **Nuevos resultados con ocrelizumab y rituximab**.

Estudio	Fármaco	Diseño	Población	Principales resultados de eficacia	Principales resultados de seguridad
ORATORIO-HAND [48]	Ocrelizumab 600 mg	Fase IIIb, doble ciego, aleatorizado con placebo	EMPP	- 30% ↓ riesgo CDP compuesta (EDSS/9 HPT) a las 12 semanas.	No hubo aumento de infecciones graves ni de tumores
			N = 1013; 505 ocrelizumab, 508 placebo		
				- 33% ↓ riesgo CDP (EDSS) a las 12 semanas.	
			- 27% >55 años		
			- 24% Gd+	- 41% ↓ riesgo CDP (9 HPT) a las 12 semanas.	
			- $\bar{x}$ EDSS 6		
GAVOTTE [49]	Ocrelizumab 1200/1800 mg	Fase IIIb, doble ciego, aleatorizado con ocrelizumab 600 mg	EMPP	Una dosis más elevada de ocrelizumab no logra reducir la progresión de la discapacidad, medida por EDSS, 9 HPT, y T25-FW	- No hubo diferencias entre dosis normales y elevadas.
			N = 753; 500 ocrelizumab 1200/1800 mg, 253 ocrelizumab 600 mg		
					- Incidencia de tumores similar a las cohortes observacionales y población general.
			- $\bar{x}$ 43 años		
			- 26% Gd+		
OPERETTA 2 [50]	Ocrelizumab (300/600 mg) vs Fingolimod (0,25/0,5 mg)	Fase III aleatorizado, doble ciego y doble simulación	EMRR	Ocrelizumab ↓45% la TAB	Ligero aumento de infecciones con ocrelizumab.
			N = 187; 93 ocrelizumab, 94 fingolimod	Ocrelizumab ↓48% las lesiones en T2	
			- $\bar{x}$ 15 años	Ocrelizumab ↓87% las lesiones en T1 Gd+.	
			- TAB = 1.4		
			- ≈50% Gd+		
RIDOSE-MS [51]	Rituximab 500 mg cada 6 meses *vs* rituximab cada 12 meses	Fase III	EMRR	NEDA-3 a los años 2–4 es similar en ambos regímenes	El efecto en los niveles de inmunoglobulinas y la tasa de infecciones fue similar.
			N = 206; 103 rituximab cada 12 meses, 103 rituximab cada 6 meses		
			- 97% sin brotes		
			- $\bar{x}$ EDSS 1		

9 HPT, prueba de los 9 agujeros (*Nine-Hole Peg Test*); CDP, progresión confirmada de la discapacidad; Gd, gadolinio; EMPP, esclerosis múltiple primaria progresiva; EMRR, esclerosis múltiple remitente-recurrente; NEDA, no evidencia de actividad de la enfermedad; T25-FW, prueba cronometrada de 25 pies (*Timed 25-Foot Walk*); TAB, tasa anualizada de brotes. Las flechas (↓) indican disminución.

## 8. Expectativas de Tratamiento en Enfermedades Relacionadas

En el trastorno del espectro de la neuromielitis óptica (NMOSD, neuromyelitis optica spectrum disorder), las nuevas dianas terapéuticas incluyen CD38 (daratumumab), células plasmáticas (bortezomib), factor activador de linfocitos B/ligando inductor de proliferación (BAFF/APRIL) (telitacicept), inhibidores de FcRn (batoclimab y efgartigimod), BTKi (zanubrutinib) y acuaporina 4 (AQP4) (aquaporumab) [52]. El uso de células CAR-T empieza a dar buenos resultados con la mayoría de los pacientes participantes en un estudio fase 1 libres de brotes [53].

La enfermedad por anticuerpos contra la glicoproteína de la mielina de los oligodendrocitos (MOGAD, *myelin oligodendrocyte glycoprotein antibody-associated disease*) es recurrente en el 70% de pacientes a los 5 años, aunque actualmente no hay evidencia de clase I que respalde ninguna estrategia terapéutica. En fases iniciales, el curso de la discapacidad es comparable al de NMOSD, mientras que a largo plazo se asemeja al de la EM. El uso de corticosteroides a los 3 meses del brote consigue reducir hasta en un 80% la tasa de recurrencias [54]. Asimismo, la administración de inmunoglobulinas [55] y plasmaféresis [56] mejoran la EDSS, y el tratamiento con tocilizumab mantiene libres de brotes al 79% de pacientes [57].

Ravulizumab, un inhibidor del epítopo C5 similar a eculizumab, es otro tratamiento emergente que ha logrado eliminar completamente los brotes en todos los pacientes del estudio CHAMPION-NMOSD tras 3 años de seguimiento [58].

Otros fármacos actualmente en investigación fase 3 en MOGAD son satralizumab (NCT05271409), rozanolixizumab (NCT05063162), azatioprina (NCT05349006) y tocilizumab (NCT06452537).

## 9. Riesgos de las Terapias de Muy Alta Eficacia

Desde el año 2022, casi el 50% de los pacientes con EM reciben terapias de muy alta eficacia en primera línea. El uso extendido de estos tratamientos, unido al riesgo potencial de infecciones en población vulnerable (edad avanzada, mayor discapacidad, niños, embarazo) requiere la implementación de estrategias de prevención [59], siendo la vacunación un elemento esencial en el manejo de estos pacientes, porque además no aumenta el riesgo de brotes [60].

Los pacientes candidatos a terapia de alta eficacia deben vacunarse previamente al inicio del tratamiento debido a la baja respuesta inmunológica a la vacunación durante dichos tratamientos [61]. Un hallazgo importante es que el número de dosis de la vacuna contra la hepatitis B administradas antes de iniciar terapia anti-CD20 aumenta la seroprotección [62]. Uno de los algoritmos de actuación que se plantean para la inmunización de pacientes candidatos a terapia de alta eficacia es hacer una terapia puente con natalizumab [63].

También se insistió en que no todos los pacientes son candidatos a terapia de alta eficacia, ya que el riesgo de experimentar acontecimientos adversos suele asociarse a factores individuales, y no solo al tiempo acumulado de tratamiento [64]. El dilema de la seguridad se hace más presente en los pacientes mayores de 55 años en quienes la discontinuación o desescalada terapéutica puede ser una opción si llevan más de 5 años sin brotes y sin discapacidad (regla del 5: 55-5-5) [65]. El aumento del riesgo de infección asociado a los fármacos anti-CD20 es evidente, de ahí la creciente evidencia sobre estrategias de minimización de riesgos. El hecho, cada vez más evidente de que extender la dosis de natalizumab reduce la seroconversión del virus John Cunningham (JC) [66], plantea la pregunta de por qué no aplicar esta estrategia a los agentes anti-CD20. Sin embargo, en el estudio RIDOSE-MS, la hipótesis de expandir las dosis de rituximab para minimizar el riesgo de infecciones no se cumplió [51]. Otro hallazgo importante proviene de los datos de seguridad de una base poblacional de Cleveland con 2500 pacientes, que ha demostrado un riesgo de infecciones graves tres veces superior con ocrelizumab respecto a los fármacos plataforma en poblaciones comparables tras el emparejamiento, sobre todo en pacientes mayores de 55 años, y un tiempo hasta la primera infección más breve [67].

## 10. Lo Más Destacado Sobre Cladribina

En el sistema nervioso central (SNC), cladribina comprimidos depleciona de forma continua una subpoblación de linfocitos B de memoria proinflamatorios de la zona marginal del bazo y favorece la repoblación de linfocitos B naive, que reaparecen aproximadamente al tercer año [68]. La detección de cladribina en el LCR el mismo día de la última dosis confirma su penetración a través de la BHE y rápida eliminación [69]. Esto se traduce en los efectos centrales que se observan a los 4 años: reducción de PIRA del 10,8% y RAW del 6,3% y una tasa de pérdida de volumen cerebral inferior al 0,4% (estimado en población general), independientemente del tratamiento previo [70,71]. El patrón de evolución de los niveles de NfL durante 4 años muestra una clara reducción sostenida en pacientes con valores iniciales >10 pg/mL, y unos niveles bajos sin aumentos en aquellos con niveles <10 pg/mL [72]. El aumento moderado que se observa al final del cuarto año puede justificar el volver a tratar con cladribina comprimidos [72].

La Tabla 2 (Referencias en **Tabla Suplementaria 1**) resume los resultados más destacados sobre el efecto de cladribina en población mayor de 50 años y en el control sostenido de la enfermedad, procedentes de cohortes de vida real nacionales e internacionales. En pacientes mayores de 50 años, destaca la baja tasa de discontinuación en comparación con la población más joven y una repoblación linfocitaria similar, junto con un buen perfil de seguridad con ningún caso de linfopenia grado 4. La evidencia en vida real a largo plazo demuestra que cladribina comprimidos es efectiva más allá de 4 años, con porcentajes elevados de pacientes libres de brotes y de actividad radiológica, tasas elevadas de persistencia y un perfil linfocitario que recupera los niveles basales tras ciclos adicionales de tratamiento.

**Tabla 2.** **Nuevos resultados de cladribina en población mayor de 50 años y control sostenido de la enfermedad**.

Estudio (Referencias en **Tabla Suplementaria 1**)		Tiempo de seguimiento; N	Principales hallazgos
Evidencia en población ≥50 años			
	MSBase	6 años	• Tasa anual de discontinuación: 4%
		N = 993	• Tasa de CDP: 6%
	Serie de Sevilla	5 años	• NEDA (años 1–4): 80%
		N = 48	• Reducción en la TAB de 0,22 a 0,013
			• Perfil de seguridad acorde a lo descrito en la práctica clínica
	Registro sueco	2,5 años (29 meses)	• Tasa de discontinuación por falta de eficacia en pacientes ≥50 años (0%) *vs* <50 años (9,9%)
		N = 392 (16% ≥50 años)	
			• La EDSS basal (1,5 <50 años y 2,9 ≥50 años) se mantuvo estable en ambos grupos.
			• Reducción de la TAB en ambos grupos: ≥50 años (0,17 año 1 *vs* 0 año 3); <50 años (0,21 año 1 *vs* 0,01 año 3).
			• Reducción de nuevas lesiones en T2 en ambos grupos: ≥50 años (9% año 1 *vs* 0% año 3); <50 años (10% año 1 *vs* 4% año 3).
			• Repoblación linfocitaria similar.
	Serie alemana	24 meses ($\bar{x}$ 16 meses)	• TAB: 0,04 (0,06 en naive, 0,02 en terapias plataforma y 0,04 en TAE).
		N = 95, estratificados según el tratamiento previo (pacientes naive, pacientes en terapias plataforma y pacientes en terapias de alta eficacia [TAE]).	
			• RAW: 0, independientemente del tratamiento previo.
			• PIRA: 0,07 (0,04 en naive, 0,09 en terapias plataforma y 0,08 en TAE).
			• Repoblación linfocitaria similar a la observada en otras cohortes.
			• Ningún caso de linfopenia grado 4, y pocos casos de linfopenia grado 3.
Evidencia más allá del año 4			
	Serie de Tenerife	Hasta 6 años	• 99 completan la pausa posológica
		N = 123	• 34 tienen un seguimiento >4 años
			• 9/34 (26%) necesitaron retratamiento: 6 en año 5 y 3 en año 6 debido a actividad clínica (n = 1) y radiológica (n = 8)
			• Causas para el retratamiento en pacientes estables que llegan al año 5: síntomas inespecíficos, presencia de factores de mal pronóstico, o por calendario
	Serie de Madrid	Hasta 7 años	• 59 pacientes (45%) están entre años 5 y 7
		N = 131	• 60% han recibido 3 cursos
			• 17% han recibido 5 cursos
			• 40% están sin tratamiento en año 6
			• Principales razones para retratar en el 42% de los pacientes: síntomas inespecíficos, factores de mal pronósticos, o deseo del paciente
	MSBase	Hasta 4 años	• Tasa de persistencia: 88%
		N = 3834	• TAB: 0,14
	Cohorte multicéntrica alemana	Hasta 6 años	• Tasa de persistencia: 66%
		N = 1642	• 21% con ciclos adicionales en año 5
	Serie de Portland	Hasta 5 años	• Brotes: 0%
		N = 267 (40% >55 años)	• Sin actividad en RM: 97%
	Cohorte multicéntrica alemana	Hasta 8 años	• Porcentaje de pacientes libres de tratamiento:
		N = 125	◦ 57,6% (72/125) al año 5
			◦ 51,2% (43/84) al año 6
			• La TAB de 0,2 al año 1 se mantiene estable durante 7 años de seguimiento.
			• La población linfocitaria tras continuación de tratamiento/ciclos adicionales recupera a niveles basales.
	Cohorte multicéntrica alemana	Hasta 8 años	• 84% de pacientes están libres de brotes tras 18 meses de la reintroducción del fármaco en año 5.
		N = 70	
			• 70% de los pacientes reciben un ciclo adicional por calendario.
			• La población linfocitaria en los ciclos adicionales sigue una evolución similar a la observada durante los 4 primeros años del inicio del tratamiento.

RAW, empeoramiento asociado a brotes; RM, resonancia magnética.

## 11. Aspectos Destacados Para la Práctica Clínica

### 11.1 Avances en el Diagnóstico de las Enfermedades Desmielinizantes: Detección más Precoz, Mayor Precisión y Ampliación del Arsenal Diagnóstico

Los nuevos criterios de McDonald 2024 [14] aumentan un 43% la probabilidad de adelantar el diagnóstico de EM una media 6 meses frente a los criterios de 2017 [73]. El porcentaje de pacientes diagnosticados se incrementa del 63% al 80%, con una reducción del tiempo diagnóstico de 84 a 40 días respecto a 2017 [74]. Estas mejoras derivan de la incorporación del síndrome radiológico aislado (RIS, *radiologically isolated syndrome*), la consideración de las cuatro topografías y la afectación del nervio óptico. No obstante, las revisiones de los criterios diagnósticos integran cada vez más pruebas paraclínicas, frente a las cuales, según una encuesta del Atlas de EM de 2024 con una participación que representa el 93% de la población mundial, pueden existir determinadas barreras de acceso, principalmente cadenas kappa, anticuerpos de tipo inmunoglobulina G contra la glucoproteína de mielina de oligodendrocitos (MOG-IgG, *myelin oligodendrocyte glycoprotein immunoglobulin G*), anticuerpos de tipo inmunoglobulina G contra la acuaporina 4 (AQP4-IgG, *aquaporin-4 immunoglobulin G*), y potenciales visuales evocados (PVE) [75].

En MOGAD, resulta especialmente relevante el desarrollo de un sistema de puntuación (TRUE-MOGAD Score), que integra variables demográficas, clínicas y radiológicas, y discrimina el diagnóstico de MOGAD con gran precisión y un área bajo la curva (AUC) superior a 0,90 [76].

Retomando el concepto de PIRA cognitivo, este fenómeno es menos frecuente en pacientes tratados con fármacos de alta eficacia [10], lo que sugiere un componente parcialmente inflamatorio. Un estudio con datos de 30.000 pacientes del registro italiano mostró que el 41,4% de los pacientes empeora por PIRA, un porcentaje que se reduce al 26,6% cuando se tienen en cuenta las lesiones subyacentes en RM [77]. Estos datos apuntan a que la inflamación probablemente desempeña un papel más importante en la enfermedad del que suele asumirse.

### 11.2 Mejora de la Evaluación del Pronóstico y la Detección de la Progresión Mediante una Mejor Comprensión de los Biomarcadores y la RM

En línea con lo previamente expuesto, los biomarcadores resultan fundamentales para diferenciar la presencia o ausencia de inflamación. Los niveles de NfL están claramente asociados al brote y la GFAP con el riesgo de PIRA. No obstante, dado que PIRA es una definición puramente clínica y los hallazgos heterogéneos acerca del papel de los NfL en su predicción, se hace necesario distinguir entre PIRA de origen inflamatorio y no inflamatorio.

En la evaluación pronóstica mediante RM, es relevante considerar el papel de las lesiones medulares. La presencia de al menos dos lesiones medulares durante el año del primer evento clínico se asocia con un mayor riesgo de progresión. Estos pacientes presentan un perfil más inflamatorio y una mayor probabilidad de requerir escalada terapéutica [78].

### 11.3 Personalización del Manejo Terapéutico mediante Nuevas Estrategias de Tratamiento

Unido a todo lo anterior, también tenemos mejoras en las opciones terapéuticas. Los datos a largo plazo obtenidos con frexalimab muestran reducciones no solo en marcadores de inflamación, como los NfL, CXCL13 o la forma soluble del marcador de linfocitos T sCD27, sino también en marcadores de activación microglial como sTREM2 [45]. Se espera que los resultados de la fase 3 confirmen si estos efectos se traducen en una disminución de la progresión de la enfermedad.

Personalizar las dosis de rituximab y extender el intervalo de infusión no parece reducir el riesgo de infección en la EM [51], aunque sí lo hace en la NMO [79]. Sin embargo, pese a la variabilidad individual, se desaconseja alargar el intervalo de infusión más allá de los 18 meses debido al aumento observado en los niveles de NfL [80].

## 12. Conclusiones

Las novedades revisadas en esta segunda parte de la XVIII Reunión Post-ECTRIMS reflejan la creciente complejidad de la esclerosis múltiple y la necesidad de abordar la enfermedad desde una perspectiva cada vez más integradora. La progresión, presente desde fases tempranas y no siempre dependiente de la actividad inflamatoria clínicamente evidente, continúa siendo uno de los principales retos clínicos y de investigación. En este contexto, el concepto de PIRA requiere una definición más homogénea y una evaluación que incorpore no solo la discapacidad física, sino también otros dominios clínicos, la resonancia magnética y los biomarcadores séricos.

Los avances en biomarcadores, especialmente NfL y GFAP, aportan información relevante sobre daño neuronal, inflamación y progresión, aunque su interpretación individual todavía presenta limitaciones. De forma paralela, la identificación y monitorización del deterioro cognitivo exigen herramientas más sensibles al cambio longitudinal, capaces de superar las limitaciones de los test actualmente utilizados en la práctica clínica y en ensayos clínicos.

La edad se posiciona como un factor determinante en la evolución de la enfermedad y en la toma de decisiones terapéuticas. El aumento de pacientes de edad avanzada, con mayor carga de comorbilidades y potencial vulnerabilidad a determinados tratamientos, obliga a individualizar el balance beneficio-riesgo, incorporar estrategias preventivas y adoptar un manejo holístico. En mujeres con EM, etapas fisiológicas como la menopausia también deben considerarse para evitar atribuir erróneamente determinados síntomas a la actividad de la enfermedad.

En el ámbito terapéutico, los tratamientos emergentes, incluidos BTKi, nuevos anticuerpos monoclonales y terapias celulares, abren nuevas perspectivas, aunque muchos resultados proceden aún de fases tempranas y requieren confirmación. Asimismo, la expansión de las terapias de alta y muy alta eficacia refuerza la importancia de la prevención del riesgo, particularmente mediante estrategias de vacunación y monitorización individualizada. En conjunto, los datos presentados apuntan hacia una medicina más personalizada, basada en una mejor caracterización diagnóstica, pronóstica y terapéutica de la EM.

## Contribuciones de los Autores

ÓF, XM, AArés, EA, YA, AAlon, RA, LB, CC, ABC, TCT, LCF, SE, LL, ML, SL, JEML, VML, EMonr, EMora, COG, JMP, LRP, ÀR, ARA: conceptualización, investigación, redacción—revisión & edición. Todos los autores leyeron y aprobaron la versión final del manuscrito. Todos los autores participaron suficientemente en el trabajo y aceptaron responsabilizarse de todos sus aspectos.

## Aprobación Ética y Consentimiento Informado

No aplicable.

## Material Suplementario

El material suplementario asociado con este artículo se puede encontrar, en la versión en línea, en https://doi.org/10.31083/RN55658.
