# Supplementary material for: XVIII Post-ECTRIMS Meeting: Review of the New Developments Presented at the 2025 ECTRIMS Congress (Part II)
Source: Rev Neurol. 2026 Aug 20;81(8):55658. [Article in Spanish] doi: 10.31083/RN55658 (PMC13541424; doi:10.31083/RN55658)
Supplement: Supplementary file 1 [file 1576-6578-81-8-55658-s1.zip › Supplementary Material.docx]

| Material suplementario | |
| --- | --- |
| Tabla suplementaria 1. Referencias de las ponencias de la Tabla 2 “Nuevos resultados de cladribina en poblaciones especiales y control sostenido de la enfermedad” | |
| Estudio | Referencia |
| *Evidencia en población ≥ 50 años* | |
| MSBASE | Spelman T, Real-world experience with cladribine tablets in older age groups in the MSBase Registry, 41st Congress of the European Committee for Treatment and Research in Multiple Sclerosis and the 30th Conference of Rehabilitation in Multiple Sclerosis. September 2025. Barcelona, Spain. |
| Serie de Sevilla | Rincón-Valencia A, Five-year analysis of cladribine use in patients over 50 years old with relapsing-remitting multiple sclerosis, 41st Congress of the European Committee for Treatment and Research in Multiple Sclerosis and the 30th Conference of Rehabilitation in Multiple Sclerosis. September 2025. Barcelona, Spain. |
| Registro sueco | Larsson V, Clinical effectiveness of cladribine tablets for patients below or above 50 years of age at treatment start in the Swedish Post-market Surveillance Study "Immunomodulation and Multiple Sclerosis Epidemiology 10" (IMSE 10), 41st Congress of the European Committee for Treatment and Research in Multiple Sclerosis and the 30th Conference of Rehabilitation in Multiple Sclerosis. September 2025. Barcelona, Spain. |
| Serie alemana | Axhausen F, The use of cladribine tablets in patients with MS beyond the age of 50: experiences from a large tertiary German centre, 41st Congress of the European Committee for Treatment and Research in Multiple Sclerosis and the 30th Conference of Rehabilitation in Multiple Sclerosis. September 2025. Barcelona, Spain. |
| *Evidencia más allá del año 4* | |
| Serie de Tenerife | Hernández M A, Real world outcomes in patients with relapsing multiple sclerosis with cladribine tablets in a large Spanish cohort, 41st Congress of the European Committee for Treatment and Research in Multiple Sclerosis and the 30th Conference of Rehabilitation in Multiple Sclerosis. September 2025. Barcelona, Spain. |
| Serie de Madrid | Oreja-Guevara C, Managing multiple sclerosis beyond year 4 after cladribine: Real-World challenges and strategies, 41st Congress of the European Committee for Treatment and Research in Multiple Sclerosis and the 30th Conference of Rehabilitation in Multiple Sclerosis. September 2025. Barcelona, Spain. |
| MSBASE | Spelman T, Real-world experience with cladribine tablets in the MSBase Registry: 2025 update, 41st Congress of the European Committee for Treatment and Research in Multiple Sclerosis and the 30th Conference of Rehabilitation in Multiple Sclerosis. September 2025. Barcelona, Spain. |
| Cohorte multicéntrica alemana | Korsukewitz C, Treatment continuation with cladribine tablets (CladT) beyond year 4– second interim analysis of the CLIP-5 study, 41st Congress of the European Committee for Treatment and Research in Multiple Sclerosis and the 30th Conference of Rehabilitation in Multiple Sclerosis. September 2025. Barcelona, Spain. |
| Serie de Portland | Smoot, Evaluating clinical outcomes and tolerability in patients 55 years and older in the providence health system who have started cladribine, 41st Congress of the European Committee for Treatment and Research in Multiple Sclerosis and the 30th Conference of Rehabilitation in Multiple Sclerosis. September 2025. Barcelona, Spain. |
| Cohorte multicéntrica alemana | Woitschach L, Retrospective analysis of multiple sclerosis patients who initiated oral cladribine 5-8 years ago - Data from a German cladribine cohort, 41st Congress of the European Committee for Treatment and Research in Multiple Sclerosis and the 30th Conference of Rehabilitation in Multiple Sclerosis. September 2025. Barcelona, Spain. |
| Cohorte multicéntrica alemana | Korsukewitz C, Treatment continuation with cladribine tablets (CladT) beyond year 4– second interim analysis of the CLIP-5 study, 41st Congress of the European Committee for Treatment and Research in Multiple Sclerosis and the 30th Conference of Rehabilitation in Multiple Sclerosis. September 2025. Barcelona, Spain. |
